# A Soft Thing

**Published:** 1896-11

**Authors:** 


A Soft Thing.—A position in the Marine Hospital Service must be a *sinecure*. The pay of a surgeon is $2,500 a year, and in all places where the surgeon is not furnished rooms, he draws $50 a month, commutation for quarters; and after he has been in the service five years he is paid ten per cent. of each years, salary over and above the five years. Let's see; a surgeon—we will say, has been, in the service say—ten years. His salary would be $2,500, plus commutation for quarters, $50 a month, $600; ten per cent of $2,500 is $250 for five years, $1,250; making a total of $4,350—to do—what in some cases, leaves time and opportunity to lecture in a college or two, to do a private practice, and to have editorial charge of a large weekly medical journal.

Assistant and passed assistant surgeons receive respectively, $1,600 and $1,800 salary, $30 and $40 per month commutation for quarters; but *sometimes* one of these gets sent to a place where things are not as lovely as they were at Chicago; young Branan, for instance, was sent to Brunswick, Ga., to put down yellow fever, in 1892, and died of the fever.

